# Hepatitis B, C, and delta in the general population in Mayotte: hepatitis B as a major public health concern

**DOI:** 10.1186/s12879-022-07679-7

**Published:** 2022-08-29

**Authors:** Cécile Brouard, Fanny Parenton, Hassani Youssouf, Stéphane Chevaliez, Emmanuel Gordien, Maxime Jean, Mathias Bruyand, Sophie Vaux, Florence Lot, Marc Ruello, Unono Wa Maore group, Marion Fleury, Jean-Baptiste Richard, Jean-Louis Solet, Laurent Filleul, Delphine Jezewski-Serra, Julie Chesneau

**Affiliations:** 1 HIV, Hepatitis B/C and STI Unit, Santé publique France, the National Public Health Agency,, Saint-Maurice, France; 2 Mayotte Regional Office, Santé publique France, the National Public Health Agency, Mamoudzou, France; 3 National Reference Centre for Viral Hepatitis B, C and Delta, Department of Virology, Henri Mondor University Hospital, Créteil, France; 4 National Reference Centre for Viral Hepatitis B, C and Delta, Department of Virology, Paris-Seine-Saint-Denis University Hospitals, Bobigny, France; 5 Mayotte Health Regional Agency, Mamoudzou, France; 6 Survey Unit, Santé publique France, the National Public Health Agency, Saint-Maurice, France; 7 La Reunion Regional Office, Santé publique France, the National Public Health Agency, Saint-Denis, France; 8 Region Unit, Santé publique France, the National Public Health Agency, Saint-Maurice, France

**Keywords:** Hepatitis B, Hepatitis C, Hepatitis delta, Prevalence, Mayotte, General population

## Abstract

**Background:**

Located in southwestern Indian Ocean, Mayotte is a French territory, with a very specific demographic, social and health context. To date, epidemiological data on infections by hepatitis B (HBV), C (HCV), and delta (HDV) viruses in Mayotte have been sparse. We aimed to estimate, in the 15–69-year-old general population living in Mayotte, the prevalence of infections by hepatitis B (HBV), C (HCV), and delta (HDV) viruses and the distribution of HBV status: current infection with positive HBs antigen (Ag); resolved infection with positive HBc antibodies and negative HBsAg; immunisation by vaccination with only positive HBs antibodies; and no infection/no immunisation with negative markers. We also described the characteristics of infected people and assessed the determinants of lifetime HBV infection.

**Methods:**

The Unono Wa Maore survey, implemented in a random sample of the general population in 2018–2019, consisted of an at-home collection of epidemiological data and venous blood samples. Detection of hepatitis B, C, and delta serological and molecular markers was performed.

**Results:**

Among 5207 eligible people, 4643 responded to the questionnaire (89.2%), with 2917 being tested for HBV and HCV (62.8%). Estimated HBV status was as follows: current infection 3.0% (95% confidence interval [CI]: 2.3–3.9%) (n = 76); resolved infection 27.8% (95% CI: 25.8–29.9); immunisation by vaccination 27.7% (95% CI: 25.9–29.7); and no infection/no immunisation 41.5% (95% CI: 39.3–43.7). One participant was positive for HDV antibodies (Ab) (0.65%) with a negative HDV-RNA viral load. The risk of lifetime HBV infection was higher in men (adjusted prevalence ratio (aPR): 1.55, 95% CI: 1.29–1.89); in people aged 30–49 years (aPR: 3.83, 95% CI: 1.49–9.81) or 50–69 years (aPR: 4.52, 95% CI: 1.77–11.53) compared to those under 20; in individuals who reported no condom use during their first sexual intercourse (aPR: 1.46, 95% CI: 1.01–2.14); and in those living in Dembeni-Mamoudzou (aPR: 1.40, 95% CI: 1.09–1.80) compared to the West-Centre of Mayotte. Finally, six individuals were positive for HCV antibodies (0.21%), including three positive for HCV RNA.

**Conclusions:**

Mayotte is an area of intermediate endemicity for HBV and low endemicity for HCV and HDV. With a prevalence of HBsAg 10 times higher than in mainland France, a high proportion of people susceptible to HBV infection, and a demographic, health, and social context that may favour its transmission, hepatitis B is a major public health concern in Mayotte.

## Background

Located in the Comoros archipelago in the southwestern Indian Ocean, Mayotte is the smallest French territory (376 km^2^), though with the highest population density (768 inhabitants/km^2^) after Ile-de-France (Paris region) [1]. Estimated at 288,926 inhabitants (on January 1, 2021), the population is very young, with 53.8% being under 20 years [1]. Mayotte has experienced strong demographic growth (+ 3.8% on average per year since 2012) [2], mainly linked to a very high birth rate (35.2‰ vs 10.7‰ for mainland France) [3] and high levels of immigration, mainly from the Comoros [2]. Consequently, almost half of the population living in Mayotte (48%) is of foreign nationality [2]. The social situation is quite unfavourable, with 77% of the population living below the poverty line (vs 14% in mainland France), often in precarious housing conditions (60% of dwellings lack running water, toilets, and showers) [4]. The health context is also worrying with limited health care services (e.g., the density of general practitioners is six times lower than elsewhere in France [5]), in a context marked by high frequencies of chronic diseases (especially, cardiovascular) [6, 7] or infectious diseases [8, 9] as well as insufficient vaccine coverage [10].

To date, epidemiological data on hepatitis B and C in Mayotte have been sparse and tend to focus on specific populations. The prevalence of hepatitis B surface antigen (HBsAg), indicating current infection with the hepatitis B virus (HBV), was estimated between 2.3% and 4.8% in pregnant women according to various studies carried out between 2008 and 2016 [11–14], i.e., a prevalence three to six times higher than estimated in the same population in France in 2016 (0.84%) [12]. HBsAg prevalence was 4.3% among patients hospitalised in Mamoudzou Hospital Centre (CHM) in 2014–2015 [15]. In 2016, the positivity rate of HBsAg tests performed at CHM was 3.8% vs 0.8% in France [16]. For hepatitis C virus (HCV), among 697 patients hospitalised at CHM in 2014–2015, seven (1%) had HCV antibodies (HCV Ab), of which three had a current infection (positive HCV-RNA) [15]. The positivity rate of HCV Ab tests performed at the CHM was 0.03% vs 0.7% in France in 2016 [16]. For hepatitis delta virus (HDV), there are no published data.

In the 15–69-year-old general population living in Mayotte, our objectives were as follows: (1) to estimate the prevalence of infections by HBV, HCV, and HDV and the distribution of HBV infection status according to epidemiological characteristics; (2) to describe the epidemiological and virological characteristics of infected people; and (3) to identify the socio-demographic and behavioural determinants of HBV infection.

## Methods

### Study design

We analysed the data of Unono Wa Maore, a cross-sectional health survey conducted from November 2018 to June 2019 in a random sample of the general population aged under 70 years and living in Mayotte for at least 3 months [17]. Sampling used a three-degree survey plan: random selection of 5590 geographic coordinates of the dwelling from the 2017 directory of localized buildings (inclusion of the entire territory), households (if several dwellings at the same address), and persons living in the selected household as indicated: one child under 3 years, one child under 4–14 and a maximum of three people aged 15–69 years [18]. The selected households were located based on thorough field research phase to exclude ineligible addresses (non-existent, destroyed housing, business, etc.) and to facilitate the work of investigators.

### Data collection

After obtaining informed consent, data collection consisted of face-to-face interviews performed at participants’ homes by trained investigators. Two standardised questionnaires were used for participants aged 15–69 years: a 45-min questionnaire (long questionnaire) for the first person of the household, and a short 15-min questionnaire for the other people. The data collected focussed on socio-demographic characteristics (including place of birth, educational level, life in couple), social conditions (health insurance coverage, precarious housing), perceived health condition and health situation (diet, diabetes, consumption of psychoactive substances, vector-borne diseases, etc.), recourse to health care, sexuality (lifetime sexual relations, condom use during the first sexual intercourse), preventive behaviour, and especially history of anti-HBV vaccination, HIV screening and history of HCV or HBV diagnosis. Some data, including place of birth, sexuality and health insurance coverage, were collected only in the long questionnaire.

Venous blood samples and anthropometric measurements were performed at home by a nurse in participants aged 15–69 years.

### Laboratory testing

Detection of HBsAg, total HBc Ab, HBs Ab, and HCV Ab was performed using the Architect HBsAg Qualitative II, Anti-HBc II, Anti-HBs, and Anti-HCV kits, respectively, on the Architect device (Abbott Diagnostics, Des Plaines, IL). In HBsAg positive samples, the following analyses were carried out: determination of HBe status (LIAISON HBeAg/Anti-HBe, DiaSorin), detection/quantification of HBV-DNA (Alinity HBV m, Abbott), determination of HBV genotype (phylogenetic analysis of the S/P region) for samples with a HBV viral load at least 2.5 Log IU/mL [19], detection of HDV Ab (LIAISON XL Murex Anti-HDV and/or HDV Ab-ELISA-Dia.pro), and if positive, detection/quantification of HDV-RNA and determination of HDV genotype (R0 region phylogeny, CNR Delta technique). In samples positive for HCV Ab, detection/quantification of HCV-RNA (Alinity m HCV, Abbott) and determination of HCV genotype (Sentosa SQ HCV Genoptyping Assay v2) were performed.

Detection of HIV antibodies was also carried out (Architect HIV Ag/Ab Combo), with positive samples confirmed by Western blot. The level of glycated haemoglobin (HbA1c) was measured by high performance liquid chromatography.

### Definitions

HBV infection status was classified using serological status as follows [20]: current infection, resolved infection, immunisation by vaccination, and no infection/no immunisation (Table 1). Lifetime HBV infection was defined by a current or resolved infection, that is, positive HBc antibodies regardless HBsAg. The cut-off considered for HBs antibody positivity was 10 mIU/mL.

**Table 1:** Definition of HBV infection status

HBV infection status	HBV serological markers		
	HBsAg	HBc Ab	HBs Ab
Current infection	+	±	±
Resolved infection	−	+	±
Immunisation by vaccination	−	−	+
No infection/no immunisation	−	−	−

*HBc Ab* HBc antibodies, *HBs Ab* HBs antibodies, *HBsAg* HBs antigen, *HBV* hepatitis B virus, +: positive; − negative

Obesity was defined by a body mass index of at least 30 kg/m^2^. Individuals were considered to have diabetes if a doctor had already diagnosed them with diabetes or if their HbA1c level was at least 6.5%.

Precarious housing was defined as a construction that was not solid or lacking running water or toilets in the dwelling.

In accordance with the administrative division of Mayotte, the 17 municipalities were grouped into five inter-municipalities (Dembéni-Mamoudzou, North, West-Centre, Petite-Terre, and South) [21].

### Data analysis

Data analysis concerned participants aged between 15 and 69 years. First, characteristics of participants screened for HBV and HCV were compared to those of all the survey participants. Then, analysis was restricted to screened participants.

The prevalence of HBV and HCV infections was estimated as the proportion of people who tested positive among those tested and then extrapolated to the general population living in Mayotte.

Comparisons were made using the Chi-square test for qualitative variables with a significance level of 5%.

Poisson regression models were used to assess the determinants of current HBV infection. To maximise the power, this analysis included all participants screened for HBV and was adjusted for the variables common to the long and short questionnaires. Poisson regression models were also used for lifetime HBV infection. This analysis included participants screened for HBV who had completed the long questionnaire to allow for adjustments to the variables only included in this questionnaire (e.g., place of birth, sexual behaviour).

Variables included in the multivariate models had at least one category with a *P*-value < 0.20 in univariate analysis. The threshold of 0.05 was considered statistically significant in multivariate analyses.

All the results were weighted and adjusted to take into account sampling and non-responses at both the household and individual levels [17, 18].

Analysis was performed using Stata 14.2 (StataCorp., USA).

## Results

### Participant and population characteristics

**Fig. 1:**
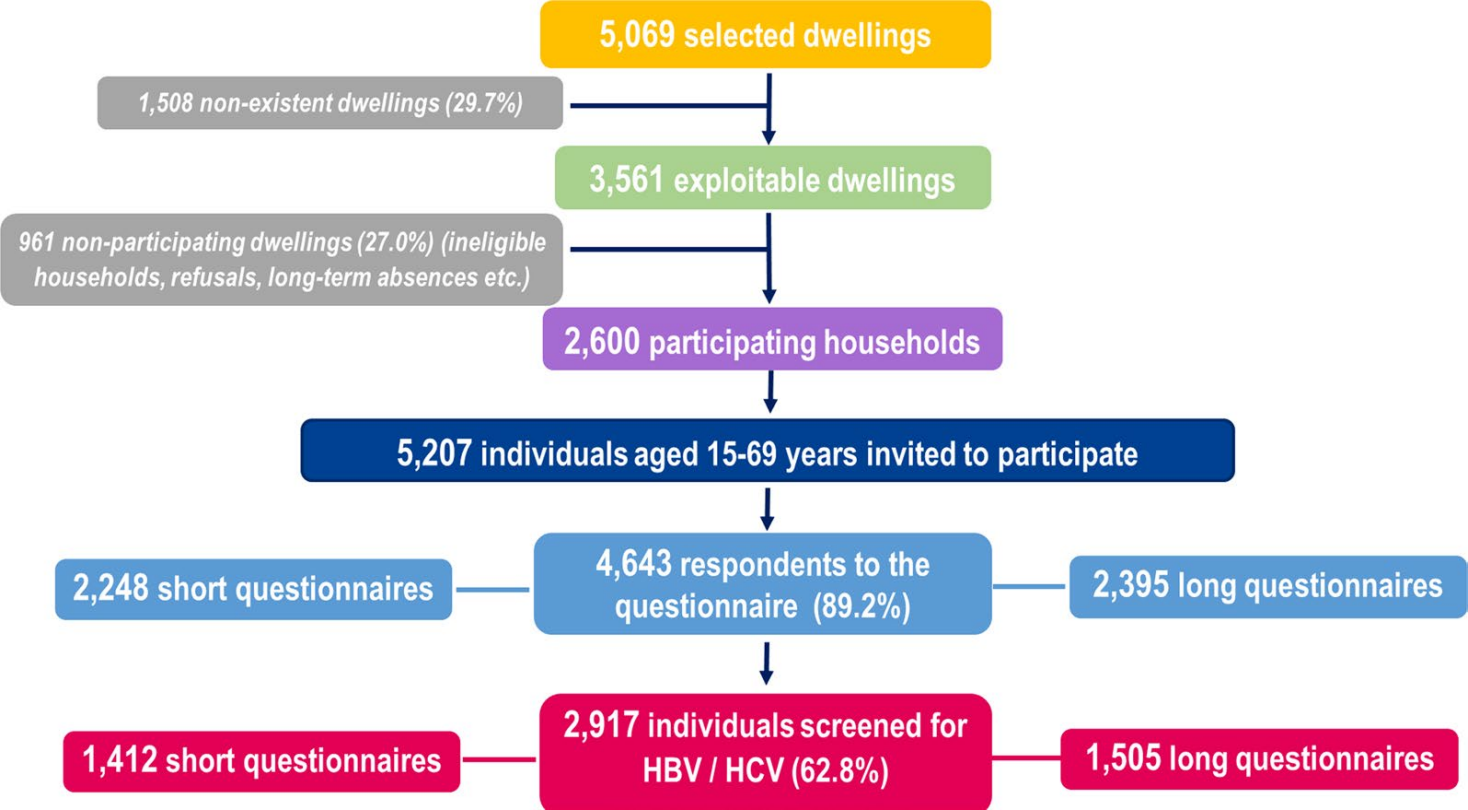
Flow-chart, Unono Wa Maore, Mayotte, 2018–2019. *HBV* hepatitis B virus, *HCV* hepatitis C virus

Among the 5069 selected households, 3561 were exploitable (70.3%) of which 2600 households (73.0%) participated in the survey (Fig. 1) [17]. In these households, 5207 people aged 15–69 years were invited to participate, with 4643 (89.2%) answering the short (n = 2248) or long (n = 2395) questionnaires. Among them, 2917 people were tested for HBV and HCV serological markers (62.8%): 1412 and 1505 of these tested participants answered the short and long questionnaires, respectively.

After weighting, the distributions of the main socio-demographic and epidemiological characteristics of participants tested for HBV and HCV were similar to those of all participants, except for place of birth, as 49.4% of screened participants were born in the Comoros compared to 46.7% of all participants (Table 2).

**Table 2:** Socio-demographic and epidemiological characteristics of participants screened for HBV and HCV compared to all participants in the Unono Wa Maore survey, Mayotte, 2018–2019

	Participants screened for HBV/HCV (n = 2917)			All survey participants (n = 4643)	
	n	Raw %	Weighted %	n	Weighted %
Gender					
Men	1074	36.8	46.0	1856	46.0
Women	1843	63.2	54.0	2787	54.0
Age (years)					
15–19	519	17.8	18.4	848	18.3
20–29	585	20.0	23.9	938	24.0
30–49	1277	43.8	43.3	2018	43.0
50–69	536	18.4	14.4	839	14.7
Place of birth^1^					
Mayotte	576	38.3	42.5	1021	45.1
Comoros	789	52.5	49.4	1134	46.7
France excluding Mayotte	32	2.1	1.8	68	2.4
Other countries	107	7.1	6.3	168	5.8
Place of residence					
Dembeni-Mamoudzou	1334	45.7	34.5	1899	34.1
North	233	8.0	20.2	650	21.9
West-Centre	571	19.6	20.5	953	19.8
Petite-Terre	474	16.2	13.4	630	12.5
South	305	10.5	11.4	511	11.7
Educational level					
No diploma	1815	63.6	60.8	2747	58.7
< Secondary school certificate	760	26.6	27.5	1262	29.2
Secondary school certificate or higher	279	9.8	11.7	515	12.1
Health insurance coverage^1^					
None	573	38.2	36.5	850	35.9
Social security only	817	54.4	56.1	1314	56.0
Social security and complementary insurance	111	7.4	7.4	217	8.1
Precarious housing					
Yes	1706	58.5	57.7	2549	57.3
No	1211	41.5	42.3	2094	42.7
Living in couple					
Yes	1649	56.6	57.2	2640	57.4
No	1267	43.4	42.8	1999	42.6
Lifetime sexual relations^1^					
Yes	1318	88.1	82.8	2087	82.9
No	178	11.9	17.2	293	17.1
Condom use during first sexual intercourse^1,2^					
Yes	172	15.6	18.8	294	19.5
No	927	84.4	81.2	1445	80.5
Perceived health condition					
Excellent/very good/good	1339	46.9	48.8	2215	50.1
Intermediate	1015	35.6	34.8	1572	34.4
Poor/very poor	498	17.5	16.4	749	15.5
Reported HBV vaccine status					
Vaccinated	892	30.6	32.1	1500	33.0
Not vaccinated	407	13.9	14.5	651	14.6
Not specified	1618	55.5	53.4	2492	52.4

^1^These questions were only included in the long questionnaire (n = 1505 for participants screened for HBV/HCV; n = 2395 for survey participants aged between 15 and 69 years)

^2^Among individuals who reported having sexual relations in their lifetime

### Hepatitis B and delta

**Fig. 2:**
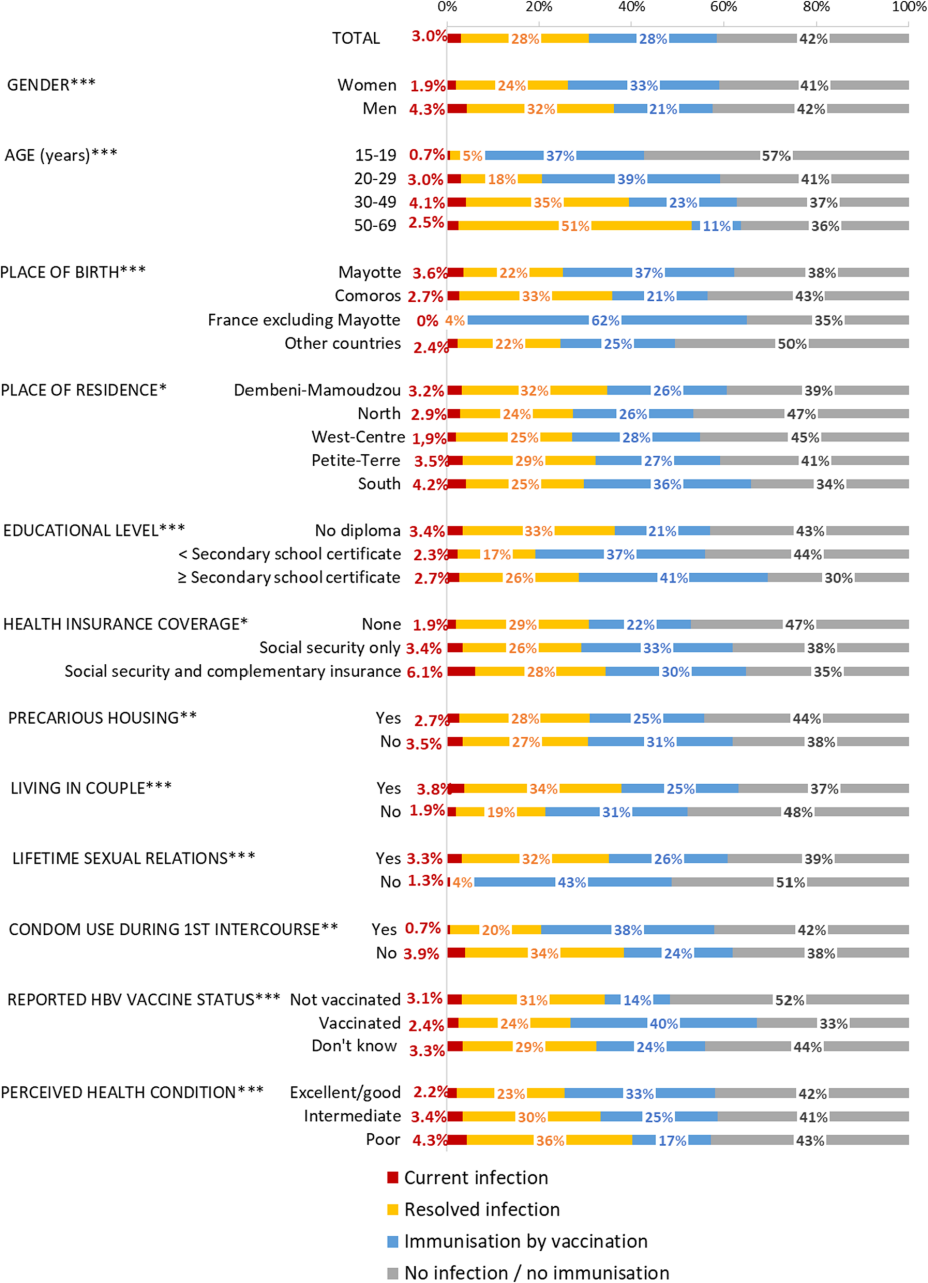
HBV virological status in the 15–69-year-old general population living in Mayotte, Unono Wa Maore, 2018–2019. *P value < 0.05; **P value < 10^–2^; ***P value < 10^–3^ for the Chi-2 test comparing HBV infection status (four modalities) according to the different qualitative variables. Only the long questionnaire included the variables of place of birth, health insurance coverage, lifetime sexual relations, condom use at first sexual intercourse, and perceived health condition

HBV serological status was available for 2916 participants (HBsAg result was missing for one person). HBV infection status was significantly associated with all socio-demographic and epidemiological characteristics studied in univariate analysis (Fig. 2).

#### Current HBV infection

Overall, 76 participants tested positive for HBsAg, corresponding to an estimated prevalence of current HBV infection of 3.0% (95% confidence interval [CI]: 2.3–3.9) in the general population aged 15–69 years and living in Mayotte (Fig. 2). The prevalence was twice as high in men than in women (4.3% vs 1.9%, P < 10^–2^) and was highest in those aged 30–49 years (4.1%). It was significantly higher in people living in couples (3.8% vs 1.9%, P < 10^–2^) and in those who declared no condom use during their first sexual intercourse compared to those who indicated using one (3.9% vs 0.7%, P < 0.05).

In multivariate analysis, men had an increased risk of having a current HBV infection [adjusted prevalence ratio [aPR]: 2.35 (95% CI: 1.38–4.00)], as did individuals aged 20–29 [aPR: 3.89 (95% CI: 1.21–12.55)] or 30–49 years [aPR: 4.83 (95% CI: 1.48–15.74)] compared to those aged under 20 (Table 3).

**Table 3:** Univariate and multivariate analysis of current HBV infection (positive HBsAg) according to socio-demographic characteristics in the 15–69-year-old general population living in Mayotte, Unono Wa Maore, 2018–2019 (n = 2916^1^)

	Univariate analysis			Multivariate analysis		
	PR	95% CI	*P* value	aPR	95% CI	*P* value
Gender						
Women	1.00			1.00		
Men	2.33	1.37–3.96	0.002	**2.35**	**1.38–4.00**	**0.002**
Age (years)						
15–19	1.00			1.00		
20–29	0.99	0.50–1.96	0.980	**3.89**	**1.21–12.55**	**0.023**
30–49	1.94	1.11–3.41	0.021	**4.83**	**1.48–15.74**	**0.009**
50–69	0.82	0.36–1.85	0.628	2.81	0.67–11.71	0.156
Place of residence						
West-Centre	1.00					
Dembeni-Mamoudzou	1.08	0.65–1.80	0.756			
North	0.96	0.40–2.27	0.919			
Petite-Terre	1.19	0.63–2.25	0.598			
South	1.47	0.62–3.47	0.377			
Educational level						
No diploma	1.00					
< Secondary school certificate	1.45	0.80–2.65	0.222			
≥ Secondary school certificate	1.15	0.45–2.95	0.769			
Precarious housing						
No	1.00					
Yes	0.77	0.45–1.32	0.348			
Living in couple						
No	1.00			1.00		
Yes	2.04	1.18–3.51	0.010	1.32	0.69–2.50	0.402

^1^HBsAg result missing for one person

*aPR* adjusted prevalence ratio, *PR* prevalence ratio, *CI* confidence interval

Numbers in bold indicate the significant associations in multivariate analysis

Epidemiological and virological characteristics as well as comorbidities of people with a current HBV infection are presented in Table 4.

**Table 4:** Epidemiological characteristics, coinfections and comorbidities of HBsAg positive people (compared to those negative) in the 15–69-year-old general population living in Mayotte, Unono Wa Maore, 2018–2019

	HBsAg positive		HBsAg negative		P value*
	n	%	n	%	
Total	**76**	**100**	***2840***	**100**	
Epidemiological characteristics					
Men	**42**	**66.5**	***1031***	***45.3***	***0.001***
Age (years)					
15–19	**4**	**4.4**	***5155***	***18.8***	***0.021***
20–29	**17**	**23.8**	***68***	***24.0***	
30–49	**46**	**59.8**	***1231***	***42.8***	
50–69	**9**	**12.0**	***526***	***14.4***	
Place of birth^1^					
Mayotte	16	50.8	*560*	*42.2*	*0.743*
Comoros	16	44.3	*773*	*49.6*	
France excluding Mayotte	0	0	*32*	*1.8*	
Other countries	2	4.9	*105*	*6.4*	
Educational level					
No diploma^2^	50	69.1	*1827*	*61.4*	*0.460*
< Secondary school certificate	19	20.7	*741*	*27.1*	
Secondary school certificate or higher	7	10.2	*272*	*11.5*	
Health insurance coverage^1^					
None	8	22.8	*565*	*36.9*	*0.244*
Social security only	22	62.3	*795*	*55.9*	
Social security and complementary insurance	4	14.9	*107*	*7.2*	
Precarious housing	38	51.4	*1668*	*57.9*	*0.346*
Living in couple	**52**	**73.1**	***1596***	***56.7***	***0.009***
Lifetime sexual relations^1^	31	92.3	*1287*	*82.6*	*0.199*
Condom use during the first sexual intercourse^1,3^	**2**	**4.1**	***170***	***19.3***	***0.014***
Perceived health condition					
Excellent/very good/good	30	35.6	*1309*	*49.2*	*0.126*
Intermediate	27	40.5	*988*	*34.7*	
Poor / very poor	17	23.9	*480*	*16.1*	
Coinfections and comorbidities					
HCV Ab positive	0	0	*6*	*0.21*	*0.726*
HIV Ab positive	0	0	*3*	*0.1*	*0.785*
HDV Ab positive	1	0.65	*–*	*–*	*–*
Obesity	25	29.8	*885*	*27.8*	*0.739*
Diabetes	11	13.6	*337*	*10.8*	*0.489*

*Chi-2 test; the distributions are significantly different for numbers in bold

*Ab* antibodies, *HBeAg* HBe antigen, *HBsAg* HBs antigen, *HBV* hepatitis B virus, *HCV* hepatitis C virus, *HDV* hepatitis D virus

^1^These questions were only included in the long questionnaire (n = 1505)

^2^People who answered “other” or did not answer were grouped with those who answered “no diploma.”

^3^Among individuals who reported having sexual relations in their lifetime

Almost 30% were obese and 13.6% presented diabetes, although these comorbidities were not related to HBsAg status. None were co-infected with HIV or HCV. Almost all were HBeAg negative (93.5%). The HBV-DNA viral load (detectable for 99%) was less than 2000 IU/ml, between 2000 and 20,000 IU/mL, and greater than 20,000 IU/mL for 64.0%, 23.2%, and 11.8% of people, respectively (Table 5). From the 47 samples for which HBV genotyping could be performed, the HBV genotypes were A (69.4%) and D (30.6%) (genotyping failure in the remaining 29 HBsAg positive people due to undetectable or too low viral load). Only one person was positive for HDV antibodies (0.65%), with an undetectable RNA HDV. Twelve participants reported that a doctor had diagnosed them with hepatitis B among the 34 HBsAg (+) individuals (32.1%) who answered the question in the long questionnaire.

**Table 5:** Virological characteristics of HBsAg positive people in the 15–69-year-old general population living in Mayotte, Unono Wa Maore, 2018–2019

	n	%
Total	76	100
HBV-DNA viral load (n = 75)*		
Not detectable	1	1.0
Detectable but not quantifiable	4	7.3
< 2000 UI/mL	45	56.7
2000–19,000 UI/mL	13	23.2
≥ 20,000 UI/mL	12	11.8
HBV genotypes (n = 47)*		
A	32	69.4
D	15	30.6
HBeAg (n = 72)*		
Positive	7	6.5

*Numbers of HBsAg positive people in whom virological analyses could be carried out

#### Resolved HBV infection

The proportion of the population living in Mayotte aged 15–69 years with a resolved HBV infection was estimated at 27.8% (95% CI: 25.8–28.9). This proportion varied significantly according to the studied characteristics with the exception of health insurance coverage, reported anti-HBV vaccination status, and precarious housing (Fig. 2). More specifically, this proportion was higher in men (31.9%) than in women (24.3%, P < 10^–3^) as well as in participants born in the Comoros (33.1%) than in Mayotte (21.5%), France (excluding Mayotte) (3.5%), or another country (22.3%). The proportion of those with a resolved HBV infection reached 50.5% among 50–69 year-old participants.

#### Lifetime HBV infection

The estimated proportion of people living in Mayotte aged 15–69 years who had been infected with HBV during their lifetime (regardless of whether the infection was resolved or current) was 30.8% (95% CI: 28.7–32.9). In multivariate analysis, the risk of lifetime HBV infection was significantly higher in men [aPR: 1.55 (95% CI: 1.29–1.86)] and in people aged 30–49 [aPR: 3.83 (95% CI: 1.49–9.81)] or 50–69 years [aPR: 4.52 (95% CI: 1.77–11.53)] compared to those aged under 20 (Table 6).

**Table 6:** Univariate and multivariate analysis of lifetime HBV infection (resolved or current infection) according to socio-demographic and epidemiological characteristics in the 15–69 year-old general population living in Mayotte, Unono Wa Maore, 2018–2019 (analysis restricted to respondents to the long questionnaire, n = 1505)

	Number of people with lifetime HBV infection	Univariate analysis			Multivariate analysis		
		PR	95% CI	*P* value	aPR	95% CI	*P* value
Total	491						
Gender							
Women	304	1.00			1.00		
Men	187	1.58	1.30–1.91	0.000	**1.55**	**1.29–1.86**	**0.000**
Age (years)							
15–19	10	1.00			1.00		
20–29	59	0.55	0.40–0.76	0.000	2.05	0.80–5.25	0.136
30–49	265	1.83	1.51–2.23	0.000	**3.83**	**1.49–9.81**	**0.005**
50–69	157	1.79	1.48–2.17	0.000	**4.52**	**1.77–11.53**	**0.002**
Place of birth							
Mayotte	169	1.00			1.00		
Comoros	284	1.48	1.21–1.80	0.000	1.13	0.92–1.41	0.249
France (excluding Mayotte) or other countries	37	0.65	0.43–0.97	0.036	**0.57**	**0.37–0.86**	**0.009**
Place of residence							
West-Centre	87	1.00			1.00		
Dembeni-Mamoudzou	241	1.30	1.07–1.57	0.008	**1.40**	**1.09–1.80**	**0.008**
North	38	0.74	0.52–1.04	0.081	1.03	0.70–1.53	0.870
Petite-Terre	78	1.20	0.94–1.53	0.138	1.31	0.98–1.74	0.066
South	47	0.88	0.62–1.26	0.487	1.09	0.76–1.57	0.643
Educational level							
No diploma	374	1.00			1.00		
< Secondary school certificate	75	0.53	0.40–0.71	0.000	0.99	0.76–1.31	0.986
≥ Secondary school certificate	42	0.86	0.62–1.20	0.377	1.06	0.77–1.46	0.718
Precarious housing							
No	199	1.00					
Yes	292	0.96	0.79–1.17	0.681			
Living in couple							
No	187	1.00			1.00		
Yes	304	1.88	1.52–2.34	0.000	0.99	0.81–1.21	0.929
Condom use during first sexual intercourse							
Yes	30	1.00			1.00		
No/not specified	453	3.22	2.19–4.72	0.000	**1.46**	**1.01–2.14**	**0.046**
No sexual relations in the lifetime	8	0.65	0.43–0.99	0.044	0.56	0.21–1.49	0.242

*aPR* adjusted incidence rate ratio, *PR* incidence rate ratio, *CI* confidence interval

Numbers in bold indicate the significant associations in multivariate analysis

In univariate analysis, individuals born in the Comoros were more likely to have been infected during their lifetime than those born in Mayotte, but this association was no longer statistically significant in multivariate analysis. The risk of lifetime HBV infection was lower for individuals born in France (excluding Mayotte) or in other countries [aPR: 0.57 (95% CI: 0.37–0.86)] compared to those born in Mayotte. People living in Dembeni-Mamoudzou [aPR: 1.40 (95% CI: 1.09–1.80)] had a higher risk of having been infected compared to those living in the West-Centre of Mayotte, as well as those living in Petite-Terre though it was not statistically significant. Regarding sexual behaviour, the risk of having been infected with HBV was significantly higher in individuals who reported not using a condom during their first sexual intercourse [aPR: 1.46 (95% CI: 1.01–2.14)] compared to those who declared using one.

#### Immunisation by vaccination

The proportion of people immunised by vaccination in the 15–69-year-old population living in Mayotte was estimated at 27.7% (95% CI: 25.9–29.7) and varied significantly according to the investigated variables (Fig. 2). In particular, this proportion was higher among those with self-reported vaccination against HBV (40.5%) than those declaring to be unvaccinated (14.1%) or not knowing (23.6%). It reached 37.9% among those under 30 years.

#### No infection/no immunisation

More than four in ten people in the general adult population [41.5% (95% CI: 39.3–43.7)] were estimated to be negative for all three serological markers and therefore susceptible to infection with HBV and HDV. This proportion reached 47.9% among those under 30 years and 51.3% among those who reported no sexual intercourse in their lifetime.

### Hepatitis C

Among the 2917 people screened for HCV antibodies, only six were positive (0.21%). These three men and three women had an average age of 56.3 years (min = 33, max = 66). Three people had an active HCV infection (HCV RNA positive), with HCV RNA levels of 4.7, 5 and 5.2 Log IU/mL, respectively. Hepatitis C genotype was 1b, 3h, and 2 (not subtypable).

## Discussion

This survey conducted among a large random sample of the general population living in Mayotte enabled us to provide original and robust estimates of hepatitis B, C, and delta prevalence and the distribution of HBV infection status, to identify the determinants of HBV infection and to describe the epidemiological and virological characteristics of people infected with HBV.

Current HBV infection prevalence was estimated to be 3.0% (95% CI: 2.3–3.9) in 15–69 year-olds, corresponding to a prevalence 10 times higher than that estimated in the general population in mainland France in 2016 (0.3%) [22]. This is consistent with previous estimates that focussed on specific populations such as pregnant women (2.3–4.8%) [11–14], hospitalised patients (4.3%) [15], and people tested at the CHM laboratory (3.8%) [16] or in anonymous free testing consultations (4.5%) [14]. Our findings confirm that Mayotte is an area of intermediate endemicity for HBV. The results also highlight that men were more affected by HBV with an estimated prevalence of 4.3%, which is more than twice as high as that estimated in women (1.9%), while they also had a significantly higher risk of being infected regardless of their other characteristics. Men should therefore constitute a target population for HBV testing. Indeed, with a high fertility rate (5.0 children per woman) [2] and a high rate of prenatal screening for hepatitis B (96.4%) [12] (mandatory since 1992), HBV testing may not be a pressing issue in women. Furthermore, testing is even more important, as nearly three quarters of people testing positive for HBsAg declared that they were living with a partner, with a risk of transmission to their spouse and children. The estimated proportion of HBsAg positive people indicating that a doctor had told them that they had hepatitis B (32%) should be interpreted with caution due to the small numbers of respondents and the fact that the question may have been misunderstood during the interview. In terms of age, the highest prevalence was observed among 30–49 year-olds (4.1%), although it exceeded 2% in the other age groups except for 15–19 year-olds (0.7%). Indeed, more than 80% of 15–19 year-olds were born in Mayotte and were therefore eligible for HBV universal vaccination at birth, a policy that was implemented at CHM in 1999 and officially recommended in Mayotte in 2012 [14, 23]. They were also more likely to have benefited from HBV serovaccination recommended for newborns of mothers positive for HBsAg in Mayotte as in the whole of France, although it has been shown that this preventive strategy was not systematically implemented [12, 24]. The 20–29 age group were less likely to have benefited from these two prevention measures given that they were born before 1999 and mostly in the Comoros (almost 60%) [25]. In multivariate analysis, people of this age group were at a higher risk of having a current HBV infection compared to the youngest age group and the 30–49 age group. It should nevertheless be noted that multivariate analysis could not take into account the place of birth as it would have resulted in the loss of statistical power, since this information was only provided by people who answered the long questionnaire.

Our results suggest that the transmission modes of HBV are varied and that contamination occurs at all ages, as classically described in areas of intermediate HBV endemicity. Indeed, 1.3% of people who declared no sexual intercourse were positive for HBsAg, thus suggesting perinatal or childhood transmission. Conversely, the five times higher prevalence among people who declared not using a condom during their first sexual intercourse points toward sexual transmission. The heterogeneity of the population living in Mayotte, with more than half of adults born abroad [2], mainly in the Comoros where the health and social context is particularly unfavourable [26], also probably contributes to this variability regarding HBV transmission. Even if the economic situation is more privileged in Mayotte compared to the Comoros, it is important to note that more than a third of the population is estimated to lack health insurance coverage according our results (this proportion was 32.4% in 2019 according to the Mayotte Social Security Fund [27]). This proportion was estimated at 23% among people positive for HBsAg, with a possible impact on screening and management. It should be noted that state medical aid, a specific French health insurance coverage for irregular migrants, does not exist in Mayotte, where only legal residents can be insured. This is an issue for health care access, since half of residents of foreign nationality were in an irregular situation in 2015 [25].

In terms of comorbidities, no cases of co-infection with HIV or HCV were identified, reflecting the limited circulation of these viruses in Mayotte and more widely in the Comoros archipelago [28], probably linked to the low frequency of injecting drug use and sex between men [11]. The proportion of diabetes (14%) and obesity (30%) was high in HBsAg positive people (also in those who were negative), thus constituting additional risk factors for progression to cirrhosis or liver cancer [29]. Regarding virological characteristics, the proportion of people with HBV DNA level > 20,000 IU/mL (11.8%), positive HBeAg (6.5%) or positive HDV antibodies (0.65%) was lower than observed in patients treated in expert hepatology wards in France between 2008 and 2012 (22.2%, 12.2%, and 3.7%, respectively), as these services generally care for severe patients with more advanced liver disease [30]. The HBV genotypes identified (A and D) correspond to those circulating in Africa, especially in East Africa [31].

The proportion of people with a resolved HBV infection was estimated at 27.8% (95% CI: 25.8–29.9), increasing sharply with age to reach 51% among 50–69 year-olds. Consequently, more than three in ten people aged 15–69 years living in Mayotte have a lifetime HBV infection (resolved or current). As expected, the risk of lifetime HBV infection in multivariate analysis was significantly higher in men and in people over 30 years (compared to those under 20). More surprisingly, compared to those born in Mayotte, people born in the Comoros were more likely to have been infected during their lifetime in univariate analysis, but this association was not statistically significant after adjustment to other variables, especially gender and age group, in multivariate analysis. This could be explained by significant differences between the age and sex distributions of people born in Mayotte and the Comoros, whereas the proportion of infected people varied greatly according to gender and age group [2]. The risk of lifetime HBV infection was higher in the areas of Dembeni-Mamoudzou and Petite-Terre (though not significant for the latter) compared to the West-Centre of Mayotte in multivariate analysis. These areas are characterised by the highest proportions of people born in the Comoros (respectively 58% and 54% vs 42% in the rest of the island). After adjusting to other variables, this association, especially place of birth, suggests a higher past or current circulation of HBV in these areas, regardless of the place of birth. Finally, a significant association between condom non-use and risk of lifetime HBV infection was observed, as previously shown in pregnant women by Saindou et al. [32].

In this context of significant HBV circulation, the implementation of preventive measures, in particular vaccination, is essential. While the implementation of anti-HBV vaccination at birth [14, 23] since 1999 has made it possible to achieve high levels of vaccination coverage in children (95% in children aged 24–59 months) and adolescents (75% in 14–15 year-olds) [10], which are greater than for other vaccinations [33], HBV vaccination coverage still needs to be enhanced. Thus, only 37% of young people aged 15–19 years at the beginning of their sexual life presented a serological profile indicating immunisation by vaccination. This proportion remains insufficient even considering the possible loss of HBs antibodies, estimated to concern about 40–45% of adolescents vaccinated at birth [34]. Indeed, it has been shown that protection persists for at least 30 years or even throughout life, even in the case of disappearing HBs antibodies [35]. The determinants of immunisation by HBV vaccination, which would be useful to guide the implementation of a potential new vaccination catch-up campaign as previously performed in 2018 [33], will be the subject of a specific article.

Besides the insufficient immunisation rate, the vaccine status against HBV was poorly known by participants, since more than half of the population in Mayotte was estimated to be unaware of their HBV vaccine status. In mainland France, this estimated proportion was 7% in 2016 [22]. Among people declaring to be vaccinated, 2.4% were estimated to be HBsAg positive and therefore at risk of transmitting the infection in a context of probably insufficient preventive sexual behaviours. Thus, only 19.5% of people indicated using a condom at their first sexual intercourse. This proportion was estimated to be 35.2% among 18–29 year-olds living in Mayotte (data not shown) vs 85% in the same population in mainland France in 2016 [36].

For hepatitis C virus, only six of the 2917 people tested for HCV antibodies were positive (0.21%), including three positive for HCV RNA. This result confirms that Mayotte is a low endemic area for HCV, similarly to mainland France where the prevalence of HCV RNA was estimated at 0.3% among the general population in 2016 [22].

As the objective of the Unono Wa Maore survey was to describe the state of health and health care use for the population living in Mayotte, choices were made to limit the length of time for completing the questionnaires. Thus, the epidemiological data collected on hepatitis were limited and only appeared in the long questionnaire (e.g., questions on country of birth or sexuality). Therefore, this limited the power of the statistical analyses. Further, comparisons with the results of other health surveys performed in mainland France [22, 36] must be interpreted with caution given the methodological differences and the cultural specificities of the population living in Mayotte. Finally, due to difficulties relating to the context of the survey (Ramadan that lasted from 6th of May to 5th of June 2019, during which survey respondents no longer accepted being blood drawn), not all respondents could have a blood sample and thus be screened for HBV and HCV. However, thanks to a very high participation rate in the survey (89%), nearly 3000 people, or almost 2% of all residents aged 15–69 years, were tested for HBV and HCV. Their characteristics were close to those of all participants after weighting and adjustment. The implementation of the survey, directly in the homes of participants, also made it possible to take venous blood samples to search for numerous serological and molecular markers of hepatitis B, C, and delta.

## Conclusions

This survey conducted among a large random sample of the general adult population confirmed that Mayotte is an area of intermediate endemicity for HBV and low endemicity for HCV and HDV. With a prevalence of HBsAg 10 times higher than in mainland France, a high proportion of unimmunised people, especially young people, and a demographic, health, and social context that may favour its transmission, hepatitis B should be considered as a public health priority in Mayotte. In this perspective, implementing vaccination catch-up campaigns in adolescents and young adults, strengthening screening for hepatitis B in men, as well as promoting preventive sexual behaviours are among the priority actions to be carried out.

## Data Availability

The dataset analysed during the current study is available from the corresponding author on reasonable request. The dataset is not publicly available because this survey was funded by two different institutions.

## Abbreviations

Ab: Antibodies
CI: Confidence interval
HBsAg: HBs antigen
HBV: Hepatitis B virus
HCV: Hepatitis C virus
HDV: Hepatitis D virus
PR: Prevalence ratio

## References

[1] Institut national de la statistique et des études économiques. Estimation de la population au 1er janvier 2021. Séries par région, département, sexe et âge de 1975 à 2021: INSEE; 2021. https://www.insee.fr/fr/statistiques/1893198.

[2] Chaussy C, Merceron S, Genay V. A Mayotte, près d'un habitant sur deux est de nationalité étrangère. 2019. p. https://www.insee.fr/fr/statistiques/3713016.

[3] Institut national de la statistique et des études économiques. Taux de natalité et âge moyen de la mère à la naissance en 2020, et nombre de naissances en 2019. Comparaisons régionales et départementales: Insee; 2021. https://www.insee.fr/fr/statistiques/2012761.

[4] Merceron S. Revenus et pauvreté à Mayotte en 2018, les inégalités de niveau de vie se sont creusées 2020. https://www.insee.fr/fr/statistiques/4622454.

[5] Anguis M, Bergeat M, Pisarik J, Vergier N, Chaput H. Quelle démographie récente et à venir pour les professions médicales et pharmaceutique? Constat et projections démographiques. Paris: Direction de la recherche, des études, de l'évaluation et des statistiques 2021. 74 p. https://drees.solidarites-sante.gouv.fr/sites/default/files/2021-03/DD76_0.pdf.

[6] Solet JL, Baroux N, Pochet M, Benoit-Cattin T, De Montera AM, Sissoko D (2011). Prevalence of type 2 diabetes and other cardiovascular risk factors in Mayotte in 2008: the MAYDIA study. Diabetes Metab.

[7] Ntab B, Gandin P, Castetbon K, Sissoko D, Vernay M (2007). État nutritionnel et activité physique à Mayotte, France: premiers résultats de l'étude Nutri May 2006. Bull Epidémiologique Hebdomadaire.

[8] Sissoko D, Moendandze A, Malvy D, Giry C, Ezzedine K, Solet JL (2008). Seroprevalence and risk factors of chikungunya virus infection in Mayotte, Indian Ocean, 2005–2006: a population-based survey. PLoS ONE.

[9] Youssouf H, Subiros M, Dennetiere G, Collet L, Dommergues L, Pauvert A (2020). Rift valley fever outbreak, Mayotte, France, 2018–2019. Emerg Infect Dis.

[10] Solet JL, Baroux N, Lernout T, Filleul L, Petit A, de Montera AM (2013). Estimation of the immunization coverage in Mayotte in 2010. Open Public Health J.

[11] Saindou M, Bénet T, Troalen D, Abaine A, Voirin N, Giard M (2012). Prevalence and risk factors for HIV, hepatitis B virus, and syphilis among pregnant women in Mayotte, Indian Ocean, 2008–2009. Int J Gynaecol Obstet.

[12] Brouard C, Koenig C, Bonnet C, Blondel B, Sommen C, Lot F (2020). Prévention de la transmission mère-enfant du virus de l'hépatite B en France. Enquête nationale périnatale 2016. Bull Epidémiologique Hebdomadaire..

[13] Parenton F, Youssouf H, Mariotti É, Barbail A (2020). La situation périnatale à Mayotte en 2016: principaux résultats de l'Enquête nationale périnatale (ENP) et de son extension. Bull Epidémiologique Hebdomadaire.

[14] Muszlak M, Lartigau-Roussin C, Farthouat L, Petinelli M, Hebert JC, Santiago J (2007). Vaccination of children against hepatitis B in Mayotte, French Comoros Island. Arch Pediatr.

[15] Michaud C, Vernier M, Ahmad D, Diallo A, Millot P, Olivier S (2016). Evaluation du dépistage systématique du VIH, des hépatites B, C et de la syphilis dans un service de médecine ultramarin de juillet 2014 à juin 2015. Med Mal Infect.

[16] Pioche C, Léon L, Vaux S, Brouard C, Lot F (2018). Dépistage des hépatites B et C en France en 2016, nouvelle édition de l'enquête LaboHep. Bull Epidémiologique Hebdomadaire.

[17] Ruello M, Fleury M, Richard JB, Solet JL, Bonnave PE, Filleul L, et al. The 2019 Health Barometer in the general population of Mayotte, “Unono Wa Maore” health survey. (submitted).

[18] Ruello M, Richard JB. Enquête de santé à Mayotte 2019—Unono Wa Maore. Méthode. Saint-Maurice-France: Santé publique France; 2022. 107 p. https://www.santepubliquefrance.fr/recherche/#search=Enqu%C3%AAte%20de%20sant%C3%A9%20%C3%A0%20Mayotte%202019%20-%20Unono%20Wa%20Maore.

[19] Pallier C, Castéra L, Soulier A, Hézode C, Nordmann P, Dhumeaux D (2006). Dynamics of hepatitis B virus resistance to lamivudine. J Virol.

[20] Boyd A, Gozlan J, Carrat F, Rougier H, Girard PM, Lacombe K (2018). Self-reported patient history to assess hepatitis B virus serological status during a large screening campaign. Epidemiol Infect.

[21] Préfet de Mayotte. Les services de l’Etat à Mayotte. L’intercommunalité. https://www.mayotte.gouv.fr/Politiques-publiques/Amenagement-du-territoire-Politique-de-la-Ville-et-Cohesion-Sociale/L-intercommunalite.

[22] Brouard C, Saboni L, Gautier A, Chevaliez S, Rahib D, Richard JB (2019). HCV and HBV prevalence based on home blood self-sampling and screening history in the general population in 2016: contribution to the new French screening strategy. BMC Infect Dis.

[23] Haut Conseil de la santé publique. Avis relatif à l'adaptation des recommandations et du calndrier vaccinal du département de Mayotte. Paris: HCSP; 2012. 5 p. https://www.hcsp.fr/explore.cgi/avisrapportsdomaine?clefr=254.

[24] Richaud Eyraud E, Brouard C, Antona D, La Ruche G, Tourdjman M, Dufourg MN (2015). Dépistage des maladies infectieuses en cours de grossesse: résultats de l’enquête Elfe en maternités, France métropolitaine, 2011. Bull Epidémiologique Hebdomadaire.

[25] Marie C-V, Breton D, Crouzet M, Fabre E, Merceron S. Migrations, natalité et solidarités familiales. La société de Mayotte en plien mutation 2017. p. https://www.insee.fr/fr/statistiques/2656589.

[26] Mohamed KS, Abasse KS, Abbas M, Sintali DN, Baig M, Cote A (2021). An overview of healthcare systems in Comoros: the effects of two decades of political instability. Ann Glob Health.

[27] Caisse de Sécurité Sociale de Mayotte. Les chiffres clés de la Caisse de Sécurité Sociale de Mayotte 2019. https://www.cssm.fr/uploads/espace%20presse/Les%20chiffres%20cl%C3%A9s%202019%20de%20la%20CSSM.pdf.

[28] Dada Y, Milord F, Frost E, Manshande JP, Kamuragiye A, Youssouf J (2007). The Indian Ocean paradox revisited: HIV and sexually transmitted infections in the Comoros. Int J STD AIDS.

[29] Younossi ZM, Henry L (2021). Epidemiology of non-alcoholic fatty liver disease and hepatocellular carcinoma. JHEP Rep.

[30] Santé publique France. Données de surveillance nationale de l’hépatite B chronique à partir des pôles de référence et réseaux hépatites volontaires. https://www.santepubliquefrance.fr/maladies-et-traumatismes/hepatites-virales/hepatites-b-et-d/articles/donnees-epidemiologiques-2008-2012.

[31] Tong S, Revill P (2016). Overview of hepatitis B viral replication and genetic variability. J Hepatol.

[32] Saindou M, Voirin N, Troalen D, Abaine A, Chevallier-Queyron P, Ecochard R (2013). Socio-demographic and behavioral determinants of hepatitis B vaccination and infection in pregnant women on Mayotte Island. Indian Ocean Vaccine.

[33] Subiros M, Barbail A, Larsen C. Évaluation épidémiologique de la campagne de rattrapage vaccinal chez les enfants de moins de 6 ans à Mayotte, mai-juin 2018 Saint-Maurice: Santé publique France; 2019. http://intradoc.ansp.local/exl-php/docs/spf___recherche/27271/spf00001115__PDF.txt.

[34] Schwarz TF, Behre U, Adelt T, Donner M, Suryakiran PV, Janssens W (2019). Long-term antibody persistence against hepatitis B in adolescents 14–15-years of age vaccinated with 4 doses of hexavalent DTPa-HBV-IPV/Hib vaccine in infancy. Hum Vaccin Immunother.

[35] Haut Conseil de la santé publique. Vaccination contre l’hépatite B: problématique des non-répondeurs. Paris: HCSP; 2014. 27 p. https://www.hcsp.fr/Explore.cgi/avisrapportsdomaine?clefr=475.

[36] Bajos N, Rahib D, Lydié N. Genre et sexualité. D’une décennie à l’autre. Baromètre santé 2016. Saint-Maurice: Santé publique France; 2018. 6 p. https://www.santepubliquefrance.fr/determinants-de-sante/sante-sexuelle/documents/enquetes-etudes/barometre-sante-2016.-genre-et-sexualite.

